# Plasmodesmata-mediated intercellular signaling during plant growth and development

**DOI:** 10.3389/fpls.2014.00044

**Published:** 2014-02-17

**Authors:** Shri R. Yadav, Dawei Yan, Iris Sevilem, Ykä Helariutta

**Affiliations:** Department of Biosciences, Institute of Biotechnology, University of HelsinkiHelsinki, Finland

**Keywords:** plasmodesmata (PD), non-cell autonomous proteins (NCAP), plant development, callose, symplastic domains, size exclusion limit (SEL)

## Abstract

Plasmodesmata (PD) are cytoplasmic channels that connect neighboring cells for cell-to-cell communication. PD structure and function vary temporally and spatially to allow formation of symplastic domains during different stages of plant development. Reversible deposition of callose at PD plays an important role in controlling molecular trafficking through PD by regulating their size exclusion limit. Previously, we reported several semi-dominant mutants for *CALLOSE SYNTHASE 3* (*CALS3*) gene, which overproduce callose at PD in *Arabidopsis*. By combining two of these mutations in a LexA-VP16-ER (XVE)-based estradiol inducible vector system, a tool known as the “*icals3m *system” was developed to temporally obstruct the symplastic connections in a specified spatial domain. The system has been successfully tested and used, in combination with other methods, to investigate the route for mobile signals such as the SHR protein, microRNA165/6, and cytokinins in *Arabidopsis *roots, and also to understand the role of symplastic domain formation during lateral root development. We envision that this tool may also be useful for identifying tissue-specific symplastic regulatory networks and to analyze symplastic movement of metabolites.

## INTRODUCTION

In plants, the exchange of information between cells is essential for their growth, response to the environment and defense. During development, the transmission of positional signals between different cells, tissues and organs is required for the determination of their identities. These signals include hormones, metabolites, non-cell autonomous proteins and RNAs, which can move either through the process of exocytosis and endocytosis (apoplastic signaling) or via Plasmodesmata (PD) (symplastic signaling). PD connect the cytoplasms of plant cells and act as channels for trafficking of signaling molecules, which can pass either via simple diffusion (non-targeted movement) or by temporarily changing PD diameter (targeted movement). Here, we are discussing symplastic signaling and the role of callose during plant development and describing a tool which can be used to temporally obstruct molecular trafficking through PD in a tissue-specific manner to understand the role of symplatic communication in plant developmental processes.

## PD STATES DEFINE SPATIAL SYMPLASTIC DOMAINS

Plasmodesmata are developed across the cell walls to enable cytoplasmic connection and molecular trafficking between neighboring cells. PD channels are lined by plasma membrane at their boundaries, and the desmotubule (DT), a structure composed of compressed endoplasmic reticulum (ER), is located in the center of the pores. The region between the plasma membrane and the DT is known as the cytoplasmic sleeve (CS), which provides a major path for molecular movement through PD. A large number of different kinds of proteins associated with PD have been identified using proteomic and biochemical approaches (Bayer et al., 2006; Levy et al., 2007; Thomas et al., 2008; Simpson et al., 2009; Fernandez-Calvino et al., 2011; Ham et al., 2012; Salmon and Bayer, 2013). Some of these proteins such as PDLP1 are uniformly distributed along the plasma membrane (Thomas et al., 2008), whereas others may be specifically localized to the regional membrane microdomains (Simpson et al., 2009). Therefore, presence of membrane microdomain-associated proteins at PD raises a possibility that a special membrane microdomain is associated with PD (Raffaele et al., 2009; Mongrand et al., 2010) that may act as a sorting platform for recruitment of PD-associated proteins (Mongrand et al., 2010; Simon-Plas et al., 2011; Tilsner et al., 2011). 

PD can exist in different states depending on their permeability during plant growth and development. Closed PD do not permit any trafficking, whereas small molecules such as ions, photo-assimilates and growth regulators can diffuse through opened PD. Apart from closed/open state, PD can also be in a dilated state in different tissues to allow movement of larger molecules. A dilation state of the PD is defined by their size exclusion limit (SEL) which is the upper size limit of the molecules that can move through PD. The SEL of PD varies in different cells and tissues. For example, PD located on stele/endodermis and cortex/epidermis boundaries have SEL ~60 kDa whereas PD connecting companion cells (CC) and sieve elements (SE) generally have SEL >67 kDa (Stadler et al., 2005; Rim et al., 2011). During various stages of differentiation, a dynamic control over PD permeability allows formation of some segregated regions, called “symplastic domains” in which communication among the cells is free, while between the domains, it is restricted (Rinne and Van Der Schoot, 1998; Gisel et al., 1999). These functional domains, therefore, allow specific developmental programs to take place in restricted areas. For example, the early staged embryo constitutes a single symplast due to opened interconnection between the cells, but at the later stages of development, PD change their SEL to generate distinct symplastic domains as shown by the movements of different sized tracers (Kim et al., 2005a,b).

## SYMPLASTIC SIGNALING DURING SHOOT AND ROOT DEVELOPMENT

A large number of critical cell identity regulators, non-cell autonomous transcription factors and small RNAs have been reported to traffic between cells. The first discovered mobile regulator was *KNOTTED1 (KN1)*, which regulates formation and maintenance of the shoot apical meristem (SAM) in maize (Jackson et al., 1994; Lucas et al., 1995). Subsequently, the movement of *Arabidopsis* homologs of *KN1*, *KNOTTED*1-like homeobox protein 1/*BREVIPEDICELLUS (KNAT1/BP)* and *SHOOTMERISTEMLESS (STM)* from L1 to L2/L3 layers of the SAM was shown in *Arabidopsis* (Kim et al., 2003). Yet another homeodomain transcription factor, *WUSCHEL (WUS)* moves from the organizing center to the adjacent cells of the SAM and activates *CLAVATA 3* (*CLV3*), which inturn represses *WUS* expression with *CLV1*, forming a feedback loop to control the size of the SAM (Schoof et al., 2000; Yadav et al., 2011). 

The long distance movement of *FLOWERING LOCUS* *T (FT)* from the leaves to the shoot apex via phloem to promote *LEAFY* (*LFY*) expression is required to induce flowering (Corbesier et al., 2007; Mathieu et al., 2007). *LFY* also functions non-cell autonomously by moving to adjacent cells through PD to activate downstream target genes (Sessions et al., 2000, Wu et al., 2003). Additionally, some MADS-box transcription factors exhibit non-cell autonomous functions during floral organ patterning. *Antirrhinum *B-function factors, *DEFICIENS* (*DEF*) and *GLOBOSA (GLO)*, have been shown to exhibit regulated mobility (Perbal et al., 1996). In *Arabidopsis*, the C-function gene *AGAMOUS* (*AG*) can move from the epidermal cell layer to the subepidermal cell layer of the floral meristem through secondary PD (Urbanus et al., 2010). 

In* Arabidopsis*, the quiescent center (QC) and columella cells of the root derive from the hypophysis, and other cells develop from the embryo proper (Dolan et al., 1993). The auxin response factor *MONOPTEROS (MP)* activates the expression of *TARGET OF MONOPTEROS 7* (*TMO7*) in embryonic cells, and the *TMO7* protein moves to the hypophysis precursor to promote its asymmetric division (Schlereth et al., 2010). For continuous growth and development of the root, several signaling events that balance cell division and cell differentiation are required. *SHORTROOT* (*SHR*) is expressed in the stele cells but the protein migrates to the neighboring cell layer (the QC, the cortex/endodermal initial and the endodermis). Activation of *SCARECROW (SCR)* expression by SHR in the QC is critical for specifying the QC cells and maintaining surrounding initials (Helariutta et al., 2000; Nakajima et al., 2001). In the cortex/endodermal initials (CEIs), SHR/SCR regulates the expression of a cell-cycle regulator,* CYCLIN D6*;*1* to trigger the asymmetric cell division (Sozzani et al., 2010). *WUSCHEL-RELATED HOMEOBOX 5 (WOX5)*, is expressed in the QC and like its SAM homologue *WUS*, *WOX5* non-cell-autonomously maintains columella stem cells (CSC) in the root niche (Sarkar et al., 2007), suggesting that either WOX5 itself or its downstream components move from QC to columella initials. Additionally, *ARABIDOPSIS CRINKLY4 (ACR4)* and *CLV1* assemble into a complex to perceive the *CLAVATA3/EMBRYO SURROUNDING REGION40 (CLE40)* signal and restrict the expression of *WOX5* to control the distal root meristem (Stahl et al., 2009, 2013). Interestingly, both *ACR4* and *CLV1* can interact at PD, suggesting that they may have a role in regulating the trafficking through PD (Stahl et al., 2013). 

The radial patterning of the root vascular tissues relies on a bi-directional signaling between the stele and the endodermis. SHR protein moves from the stele into the endodermis and together with SCR it activates the expression of microRNA165/6. The miR165/6 then moves in the opposite direction into the vascular tissues and establishes a concentration gradient for their targets, the *HD-ZIP III* genes (Carlsbecker et al., 2010). The miRNA-dependent post-transcriptional regulation of *PHB* expression is required for xylem specification and pericycle differentiation and also to maintain the expression of *JACKDAW (JKD)* in the ground-tissue (GT), the endodermis and the cortex, to restrict *SHR*, and *SCR* movement (Miyashima et al., 2011).

## CALLOSE PLAYS AN IMPORTANT ROLE IN REGULATING SYMPLASTIC COMMUNICATION DURING PLANT GROWTH AND DEVELOPMENT

Symplastic communication in plants is largely regulated through a control on the SEL of PD either by developmental or environmental factors. Callose is one of these factors that play an important role in regulating inter-cellular communication through PD in a wide range of developmental and physiological processes (Chen and Kim, 2009). It is biosynthesized by callose synthases (*CALS*, also called glucan synthase-like, GSL; Verma and Hong, 2001) and dynamically deposited during cell plate formation in dividing cells, during pollen development and pollen tube growth and to some specialized cell-wall domains such as PD and sieve plates of phloem SE. Its degradation, on the other hand, is controlled by activities of callose degrading enzymes called β-1, 3-glucanases (BGs). Therefore, a balance between these metabolic enzymes regulates callose levels in plant cells (Chen and Kim, 2009; Zavaliev et al., 2011). 

Despite of a large genetic redundancy among the *CALS/GSL* members, some of these genes have been shown to be involved in specific processes. For example, *CALS10* (*GSL8*) has a role during cytokinesis, stomata patterning and ploidy consistency in gametes (Chen et al., 2009; Guseman et al., 2010; De Storme et al., 2013), whereas SE-specific gene, *CALS7 (GSL7)* is required for callose deposition at PD and the sieve plates of sieve cells (Barratt et al., 2011; Xie et al., 2011). *CALS3* *(GSL12)* has a broad expression domain in *Arabidopsis* root, and the protein is localized to the plasma membrane and PD (Vatén et al., 2011). *cals3-d *gain-of-function mutants have increased level of callose at PD, resulting in pleiotropic developmental defects (Vatén et al., 2011). Similarly, BGs are also involved in a wide range biological processes including development, stress responses and pathogen defense (Doxey et al., 2007). For example, in *Arabidopsis*, AtBG_ppap controls molecular trafficking through PD and *PdBG1/PdBG2* play an important role during lateral root (LR) development (Levy et al., 2007; Benitez-Alfonso et al., 2013). However, in tobacco, a *CLASS I BETA-1,3-GLUCANASE *(*βGLU1)* is induced during seed germination and releases them from dormancy (Leubner-Metzger and Meins, 2000, 2001). In addition to the callose synthases and glucanases, several other genes also regulate symplastic trafficking by affecting callose levels (Thomas et al., 2008; Simpson et al., 2009; Lee et al., 2011). Collectively, these studies suggest that critical level of callose is required during plant development and various environmental conditions.

## ROLE OF CALLOSE IN CELLULAR ISOLATION AND SYMPLASTIC DOMAIN FORMATION DURING DEVELOPMENT

While PD provide an important path for cell-to-cell communication, regulation of their SEL at the same time also ensures a certain level of cell individuality by restricting the diffusion of certain larger factors through PD (Oparka, 1993). Some cells become even fully symplastically isolated after differentiation either by losing their PD (e.g., guard cells) or by severely restricting molecular trafficking (e.g., root cap) through PD (Erwee and Goodwin, 1985; Palevitz and Hepler, 1985; Oparka, 1993). However, formation of symplastic domains often does not require a complete closure or loss of PD, since a temporal modulation of PD permeability can be enough for creation of these functional domains during development (Epel and Bandurski, 1990). Reversible deposition of callose provides an important mechanism of control over PD in symplasmic organization. For example, in poplar and birch SAMs, callose deposition results in a closure of PD during dormancy period, which eventually is restored by β-1, 3-glucanases during chilling-induced dormancy release (Rinne et al., 2001, 2011). During stomata patterning in Arabidopsis, callose creates a local sub-domain for stomata-specific developmental programs to take place by restricting the stomata identity factor, *SPEECHLESS (SPCH)* only to the stomata initials. In cals10 mutants, stomata are developed in clusters as a result of enhanced movement of *SPCH* to neighboring cells due to increased symplastic connectivity (Guseman et al., 2010). In Arabidopsis roots, callose level controls symplastic domains in the root meristem and LR primordia. Free GFP expressed under the phloem CC specific AtSUC2 promoter is symplastically released from the CC traffics predominantly through the SE, and diffuses freely into the root tip (Imlau et al., 1999). This diffusion of free GFP is decreased in callose accumulating gfp arrested trafficking 1 (*gat1*) and *cals3-d* mutants (Benitez-Alfonso et al., 2009; Vatén et al., 2011). *GAT1* encodes for an m-type thioredoxin that controls symplastic permeability by controlling redox regulation of callose deposition in the root meristem. In Arabidopsis LRs, the callose deposition at PD correlates with symplastic domain formation during LR primordia specification and influences the initiation and patterning of LRs (Benitez-Alfonso et al., 2013). Thus, callose-mediated regulation of SEL of PD is important for creation of symplastic domains during plant development. 

## THE *icals3m* SYSTEM; A TOOL TO CONTROL MOLECULAR TRAFFICKING THROUGH PD

Although a large number of non-cell autonomous signals that control plant development have been identified, only little is known about how these signals move between the cells. This is to an extent due to absence of any suitable tool to study molecular trafficking through PD. We have recently developed a system, *icals3m,* to obstruct trafficking through PD by over-producing callose in the vicinity of PD (Vatén et al., 2011). In this system, a tissue-specific estradiol-inducible LexA-VP16-ER (XVE) system regulates the expression of a mutant *cals3m *gene in a specified spatial domain by using either a specific promoter or an enhancer-trap line, thus enabling a temporal and spatial control on callose production (Zuo et al., 2000; Vatén et al., 2011). Interestingly, the over-produced callose is not only deposited to the neck region of PD, but also along the entire PD channels, allowing a uniform closure of the channel (Vatén et al., 2011).

## APPLICATION OF THE *icals3m* IN STUDYING INTER-CELLULAR TRAFFICKING OF PROTEINS AND SMALL RNAs

**FIGURE 1:**
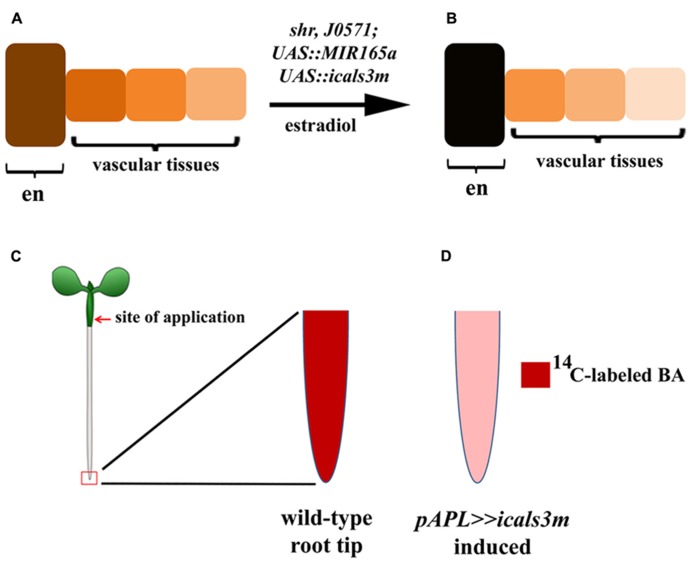
**Inducible expression of *cals3m* in ground and vascular tissues.** **(A, B)** Aniline blue stained *Arabidopsis *root tips of the *J0571; p6xUAS::icals3m* line show that upon 24 hrs 17β-estradiol treatment, callose accumulation is enhanced in the ground tissues (GT); the endodermis (en) and the cortex (co) in **(B)**. Non-induced root does not show callose in these tissues **(A)**. **(C,D)** Schematic diagram showing the use of the *icals3m *system in studying the route of SHR protein movement. **(C)** In non-induced condition, the SHR protein (green signal) is distributed in the cytoplasm of vascular tissues but after moving to the endodermis, the protein gets localize to the nucleus (N). **(D)** When *cals3m *is induced in the vascular tissues using *CRE1 *promoter, it blocks the movement of SHR protein from vascular tissues to the endodermis, suggesting that SHR protein move through PD (Vatén et al., 2011).

**FIGURE 2:**
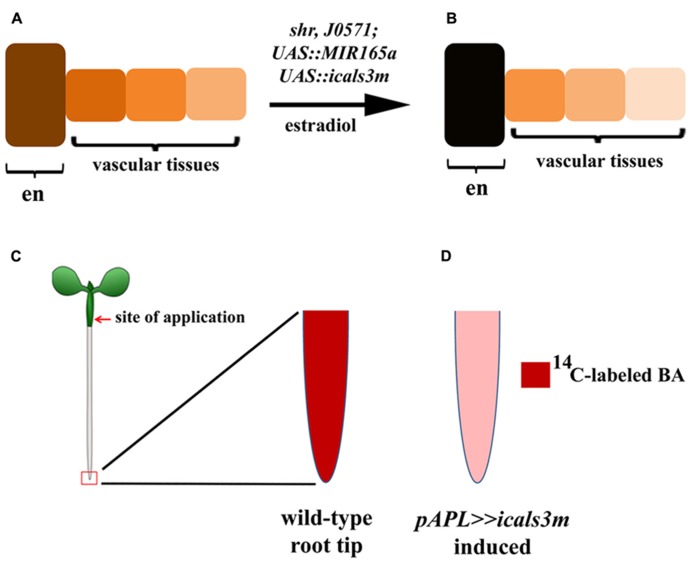
**Applications of the *icals3m *system in studying the route of miR165 and long-distance transport of cytokinins.** **(A,B)** Schematic diagram illustrating the use of *icals3m* in inhibiting the movement of miR165 from the endodermis to the vascular tissues. Only endodermis (en) of GT is shown in the schematic figure. **(A)** Under non-inducible condition, the GT expressed miR165a can diffuse freely from endodermis to the vascular tissues and makes a concentration gradient. The cells directly connected to the endodermis have high levels of miRNAs whereas miRNA levels decrease in the cells that are away from the endodermis, as shown by color gradients. (B) Upon *cals3m* induction in the GT, the miRNAs are getting trapped inside the endodermis and their concentrations in the vascular tissues are reduced. The color codes highlight miRNA concentrations in the endodermis and the vascular tissues, before **(A)** and after induction **(B)**. The miRNA signals in the endodermis of estradiol-treated plants are higher than the mock-treated plants whereas opposite pattern is seen in the vascular tissues. **(C,D)** The utility of *icals3m *in analyzing the shoot-to-root transport of cytokinins in *Arabidopsis.* ^14^C-labeled N6-benzyladenine (BA) was applied to the hypocotyls (shown by red arrow) and accumulation of the radio labeled isotope was analyzed in the root tips (highlighted by red box). **(C)** In wild-type roots, a high level of radioactive signal is seen in the root tip which is significantly reduced upon blocking the symplastic connectivity in the phloem tissues by expressing *icals3m* under *APL *promoter (**D**; Bishopp et al., 2011).

The specificity and efficiency of the system has been demonstrated in various tissues by multiple studies focusing on different biological processes. When the *cals3m* is induced in the GT-specific enhancer line (*J0571; p6xUAS::icals3m*), a high level callose is produced in the endodermis and cortex (**Figures 1A,B**), causing a hindrance in the symplastic connectivity between the endodermis and the stele, resulting in an expansion of the expression domain of *PHB *in the stele (Vatén et al., 2011). Induction of the *cals3m *in the vasculature inhibits the movement of SHR proteins from the stele to the endodermis (**Figures 1C,D**), confirming that the *SHR* protein moves via PD and that the *icals3m *system can be used to interfere intercellular protein trafficking (Vatén et al., 2011; Sevilem et al., 2013). The ability of this system to hinder the movement of miRNAs has also been analyzed. An *in situ *hybridization analysis for GT expressed *MIR165a* (*J0571; p6xUAS::MIR165a*) shows that upon *cals3m *induction in the GT (*J0571; p6xUAS::icals3m*) of *shr *mutant, the movement of *MIR165*a to the vascular tissues can be inhibited**(**Figures 2A,B**; Vatén et al., 2011)*. *This was further validated by creating a “miRNA-sensor” system by combining *icals3m *with a modified version of *MIR165A* gene, called *MIR165Amu* that is designed to target a broadly expressed nuclear-localized YFP, nlsYFP**(Miyashima et al., 2011; Vatén et al., 2011; Sevilem et al., 2013). GT-specific expression of *MIR165Amu *(*J0571; p6xUAS::MIR165Amu*)**is sufficient to remove the nlsYFP signal from the stele, however, once the movement of *MIR165Amu *is inhibited by inducing the *cals3m *in the GT (*J0571; p6xUAS::icals3m*), the nlsYFP signal re-appears in the stele. These results together suggest that *icals3m *can be effectively used to inhibit trafficking of a broad range of non-cell autonomous proteins and small RNAs.

## USING THE *ical3m* TO STUDY BIOLOGICAL PROCESSES RELYING ON SYMPLASTIC COMMUNICATIONS

In addition to its application for mobility analysis of proteins and miRNAs, Bishopp et al. (2011) used the system to elegantly demonstrate that cytokinins translocate from shoot to root via phloem. They applied ^14^C-labeled cytokinin on the hypocotyls (a shoot tissue) of wild-type, *apl *mutants that lack phloem tissues and to a transgenic line expressing *icals3m *in the phloem tissues (*pAPL::XVE>>cals3m*). The fluorescence was visualized and the radioactive signals were quantified in the root apex to analyze long-distance transport of cytokinins.**In contrast to wild-type, the basipetal transport of ^14^C-labeled cytokinins was highly compromised in the *apl *mutants and after *cals3m *induction**in the phloem of the *pAPL::XVE>>cals3m* lines (**Figures 2C,D**; Bishopp et al., 2011), suggesting that *icals3m *can also be used to hinder the long-distance transport of mobile molecules.

Moreover, apart from analyzing the mobility of a candidate molecule, the *icals3m *has also been used, as a supporting technique, in studying the significance of symplastic domain formation during LR patterning (Benitez-Alfonso et al., 2013). When *cals3m *was induced in the LR-competent xylem pole pericycle (XPP) cells using an enhancer trap-line, *J0121 *(*J0121>>cals3m*), both the LR density and positioning were affected, supporting the hypothesis that controlled intercellular symplastic connectivity among pericycle cells, founder cells and the neighboring tissues is important for *Arabidopsis* LR patterning (Benitez-Alfonso et al., 2013). This study provides an additional value to *icals3m *system that it can be applicable in interfering symplastic domain formation during organ development.

## FUTURE PERSPECTIVES

In addition to large signaling molecules (e.g., proteins and RNAs), small molecules such as nutrients and hormones also move through PD. A recent quantification of PD flux in the root meristem demonstrates that the PD flux is actually 10-fold higher than reported in an earlier study (Goodwin et al., 1990; Rutschow et al., 2011), suggesting that an efficient symplastic diffusion may be a major route for the transport of nutrients in the meristem. Interestingly, the solute flux is reduced in *Arabidopsis* line overexpressing *PDCB1*, a protein that promotes callose deposition at PD (Rutschow et al., 2011), indicating that enhanced callose deposition at PD can inhibit solute movement. Therefore, *icals3m* might be equally applicable for obstructing the symplastic movement of metabolites through PD. 

In summary, PD-mediated symplastic communication provides a major route for the movement of positional signals during plant development, and callose turnover at PD confers an important mechanism to regulate symplastic trafficking. The *icals3m *system is an effective tool to hinder symplastic trafficking through PD in a spatially and temporally regulated manner. This system has been successfully applied to inhibit movement of proteins, miRNAs, cytokinin, and to interfere a symplastic domain formation during LR development. Therefore, we envision that it can also be efficiently used for inhibiting the symplastic transport of nutrients and metabolites. Moreover, the *icals3m *system could be used widely, in combination**with other approaches, to investigate various molecular events relying on symplastic signaling.

## Conflict of Interest Statement

The authors declare that the research was conducted in the absence of any commercial or financial relationships that could be construed as a potential conflict of interest.
